# Correlation between δ^18^Ow and δ^18^Οen for estimating human mobility and paleomobility patterns

**DOI:** 10.1038/s41598-020-71683-7

**Published:** 2020-09-22

**Authors:** Elissavet Dotsika

**Affiliations:** Stable Isotope Unit, National Center of Scientific Research “Demokritos”, 15310 Ag., Paraskevi Attikis, Greece

**Keywords:** Palaeoclimate, Archaeology, Palaeontology

## Abstract

In this study a methodology for identifying the geographic origin of unidentified persons, their residence and moving patterns while providing information on lifestyle, diet and socio-economic status by combining stable isotopic data, with the biological information (isotopic composition of the skeleton), is presented. This is accomplished by comparing the oxygen isotopic composition of the spring water that individuals were drinking, during their living period, with the oxygen isotopic composition of their tooth enamel bioapatite. Spring water and teeth samples were collected from individuals from three different areas of Greece: North Greece, Central Greece and South Greece and isotopic analysis of δ^13^C and δ^18^O of tooth enamel bioapatite and δ^18^O of spring water were conducted. For these three areas the isotopic methodology is a promising tool for discriminating the provenance. Furthermore, as a case study, this methodology is applied to two archeological sites of Greece (Medieval-Thebes and Roman-Edessa) in order to determine paleomobility patterns.

## Introduction

Forensic identification of human remains is performed by various methodologies, including the examination of the biological profile, fingerprint analysis, cross reference of dental records and, in some cases, orthopedic implants and other medical devices as well as DNA analysis. Additional information about the life history of the individual before death can make the difference to a positive identification. A method capable of providing information about the residence patterns (by examining bone, teeth, hair, and nails) is the combination of oxygen isotope analysis of drinking water and of the human remains^1,2^ and can therefore complement the existing methods especially in difficult cases where only partial humans’ remains are found or the extraction of the DNA is impaired.

Stable isotopic analysis has been used (mainly the last decade) for revealing the life history of unidentified human remains^3,4^. The process is a comparative one and mainly consist of documenting the stable isotope composition of natural systems (like δ^18^O values of precipitation) and creating isotopic models^5–7^ that spatially cover the studied areas, the so called “isoscapes”. These isoscapes^2,8^ have been used in general for provenance and origin studies by comparing the unknown samples (plants, natural products, animals, soil, minerals and water) with the documented isotopic values of local samples^1,2,9–14^ and for archaeometric studies^13,15–24^. This isoscape approach can also be used to determine the geographic origin of unknown human samples (bone, teeth, hair, and nails) and to trace the residence patterns of unidentified humans^2,25–27^.

The stable isotopic composition of human tissue (C, N, H, O) will not match exactly that of the consumed food and water as various fractionation processes take place: These processes originate from the different properties of the isotopes in term of thermodynamics and kinetics but also from all the different metabolic reactions that the food undergoes during consumption^28^. Furthermore, cooking practices like boiling or treatment of the foods (preservation with salt, marinating, adding of sauces—especially fish-based) or consuming specific beverages, like milk or wine, can affect the isotopic values of human tissue in a more complicated way than just reflecting the raw food values. In addition, drinking water originating from high altitudes—like the case of Athens (~ 28 m of altitude) that is water-supplied from an altitude greater than 400 m above sea level or originating from lakes, rivers or ponds can cause a depletion or enrichment in the values of ^18^O of the local ground water, respectively and as a result complicating even further the analysis.

For the hydrogen and oxygen isotopes, their isotopic composition is greatly affected by the natural hydrological cycle. The isotopic ratios δ^2^H or δ^18^O of fresh water varies across the earth as it is affected by climatological parameters like precipitation and temperature as well as from geological parameters like altitude and continentality^29^. All the above variations are usually presented by precipitation versus stable isotope ratios plots^10,30–33^ while the spatial variations are usually displayed as isotope landscapes, or isoscapes^3,7,34^. At local scale the isotopic composition of these elements, is influenced by the movement of wet masses, their partial condensation due to the local topography and by the average rainout history^35–38^. Especially for the Mediterranean basin, the intense interactions of the sea vapors with moisture-depleted air masses of the continent can create a complicated pattern^10,39^. In general, it is observed that the warmer climates have higher δ^2^H and δ^18^O values of precipitation, while locations of higher altitude and of colder climate have lower values. The global meteoric water line is expressed by the equation: δ^2^H = 8δ^18^O + 10^32^. The meteoric line estimated for Greece is δ^2^H = 8.7δ^18^O + 19.5^10^, while the Greek spring water line is δ^2^H = 7.3δ^18^O + 8.9^11^. Despite the variability of the Greek topography, (the values of spring waters from Northern Greece are (δ^2^H = 5.8δ^18^O + 6.2), while those from Central and from South Greece are δ^2^H = 6.3δ^18^O + 0.9 and δ^2^H = 6.2δ^18^O + 6.7 respectively^11^) this equation is not so different from the global meteoric water line.

Furthermore, there is a strong seasonal and region (latitude, longitude) effect on the precipitation ^18^O values. Ground water can in general be perceived as reflecting the average δ^18^O values of the local precipitation. Contrary, springs demonstrate a lower seasonal effect on their ^18^Ο values, since mechanisms like evaporation, mixing, water flows, water–rock and water–soil interactions can have a profound effect on the ^18^O values. As a result, the water that a human consumes and affects his biocollagen or bioapatite ^18^O values can be different of the local seasonal meteoric water. This can be at the same time problematic for contemporary cases (where the drinking water is in most cases originating from a central aqueduct) but can be very helpful for archeological studies where people were drinking water from local springs. To overcome the contemporary central water supply, it is necessary to measure the ^18^O values of the local tap water, where for the archeological studies all relevant local water sources should be studied^40^.

Water undergoes a documented fractionation while it is used by biological systems (plants and animals) and can be traced by isotopic analysis of tissues (hair, tooth enamel bioapatite, or bone). After the metabolic fractionations, body water is directly related to the isotopic composition of local water^41,42^ and finally to the local climatic conditions. Furthermore, in his pioneering work, Longinelli^41^ concludes that δ^18^O_(PO4)_ values of unaltered fossil bones from domestic pigs can be used to reconstruct the δ^18^O values of local meteoric waters during the period that these mammals were living. As a result, different equations that relate the δ ^18^O of water with δ ^18^O of phosphate for different animals were proposed^40,43,44^. These equations differ according to the species. Also, in many articles, it was demonstrated that while bone and dentin apatite are frequently altered, tooth enamel bioapatite remains unaltered retaining its initial isotopic composition particularly within time scales since the Late Pleistocene and Holocene^45–47^. Consequently, the recorded signals from the biological systems are characteristic of the specific location (iso-location) from where they were recovered. Similarly, when humans move from one iso-location to another, the isotopic values of their body water are modified to reflect that of the drinking water of their current location and that can be exploited in order to identify residence patterns (assuming that the individuals were consuming tap water).

Isotopic fingerprint, in conjunction with the biological information from the skeleton, can aid in the investigation of missing persons by identifying the geographic region from which an “unidentified human” originated, including a descendant’s possible region-of-birth, long-term adult residence, recent residence/mobility patterns and dietary choices. This is accomplished by comparing the oxygen isotopic composition of the spring water that an individual was drinking, during his living period, with the oxygen isotopic composition of the body water found in bones, teeth and hair. Bones can provide a mean isotopic value of the consumed water of the last ten years. Hair can provide a record of the consumed water of the last months since a segment of hair of one centimeter long refers to roughly a month. Teeth are a record of the consumed water of early life of the individual since the tooth enamel bioapatite is formed roughly the first 20 years and remains unchanged. Furthermore, for juveniles, the tooth enamel bioapatite isotopic ^18^O values reflect the milk consumption of the nursing mother. Therefore, a sampling of different chronologically developed teeth can provide a record of the climate conditions and diet choices.

In the present study the application of this methodology to Greece is investigated. The geographic region of Greece is relative very small compared to the regions that this methodology was applied^2,8^ and there are not extreme variations in precipitation and climate. As a consequence, the spatial resolution of the isotopic values is not high and that will hinder the application of the methodology. In this framework Greece was divided in roughly three zones (North, Central and South Greece) and stable isotope analysis of teeth samples (16) of living donors that consumed tap water and seldom traveled during the early period of their life was conducted. These samples were compared to the isoscape models of δ^18^O of water from the same areas. For this analysis it was assumed that the spring and tap waters that were measured during the last decade are isotopically identical to the water these individuals consumed during their early life. This assumption is in my opinion valid since generally in Greece the consumption of bottled water is not a common practice—even today- and generally tap water originates from local spring water. Specifically in the studied areas no difference was observed between tap and local spring waters. Furthermore, a comparison was conducted of the results with the existing equations and data sets of the literature^40,41,43,44^ that correlate the oxygen isotope values of the consumed water with the oxygen isotope values of the tooth enamel bioapatite.

The methodology was applied in two case studies. The first case study with 14 contemporary tooth samples collected from the same areas but with no prior knowledge of the consumed water of the donors and the second case study with archeological tooth samples from Roman Edessa and Medieval Thebes. For the archeological samples it was assumed that the contemporary ranges of δ^18^Ow values of the spring waters (of modern Edessa and Thebes) reflect the ranges of the ancient spring waters since the climate and overall precipitation in Greece did not change drastically during the studied periods^13,48,49^.

In these case studies, the following parameters were taken in consideration: the different altitudes of the drainage basin, the temperature and the altitude of the locations as well as the altitude difference within the limits of the cities (for Roman Edessa and Medieval Thebes since there was no common water source at that era and each location within the city could be supplied by its own water source) that can affect the oxygen isotopic composition of the consumed water. The slopes of altitude versus the δ^18^Ow were calculated for the three different zones of Greece considered in this study, as well as for the areas near Edessa and Thebes in order to determine the local possible variation of the ^18^Ow values. Depending on the studied zone, a variation of 0.5 ‰ to 1 ‰ of the consumed water values is determined.

Furthermore, the effect of consuming other liquids than water (for example milk or wine) to the enamel oxygen isotopic composition was also considered. It is demonstrated that a slight variation (10%) in the liquid/water intake ratio (replacing 10% of water with liquids like milk or wine) can lead up to 1‰ variation to the derived ^18^Ow value of consumed water. The effect of nursing mothers is not considered since all non-adult samples are excluded from this study.

It is the aim of this study to demonstrate that this methodology, despite the above restrictions, is a promising tool for predicting region of origin and residence patterns of unidentified human remains and for paleomobility studies.

## Sampling and methods

In this study, the hydrogen and oxygen isotopic compositions of 75 spring waters (Table 1) were determined in order to provide the relation between δ^18^O of water (δ^18^Ow vs SMOW) and of tooth enamel bioapatite (δ^18^Oen vs PDB) of known human’s samples. These 75 spring waters were implemented to our existing database for the spring waters of Greece (containing over 450 samples)^10,11^ and produced an isoscape map slightly modified from our previous published work (Dotsika 2010 and 2018). In Fig. 2 the area of the isoscape map that is modified by the 81 spring water measurements and is relevant to this study, is presented. From these samples, 56 were used to derive equations correlating altitude and δ^18^Ow for the regions of Edessa (Pella), Thebes (Boeotia), Fhiotida, Crete and Chalkidiki in order to determine the local isotopic gradients. The altitudes for these 56 spring waters are the altitudes of the water reservoirs.

**Table 1:** Hydrogen and oxygen isotopic compositions of 75 spring waters.

n	Spring	Location	coordinates		Altitude	δ ^18^O vs SMOW	δ ^2^H vs SMOW
			Lat	Lon			
**N.Greece**							
1	Athos	Chalkidiki	40.23′	23.27′	700	− 8.2	− 54.6
2	Athos (bottled)	Chalkidiki	40.28′	23.39′	700	− 8.2	− 55.0
3	N.Triglia	Chalkidiki	40.30′	23.20′	100	− 6.1	− 43.4
4	N.Triglia	Chalkidiki	40.30′	23.20′	100	− 6.1	− 44.4
5	N.Triglia	Chalkidiki	40.30′	23.20′	100	− 6.3	− 46.9
6	N.Triglia	Chalkidiki	40.30′	23.20′	100	− 6.2	− 43.9
7	N.Triglia–N.Tenedos	Chalkidiki	40.32′	23.25′	200	− 7.1	− 48.5
8	N.Triglia–N.Tenedos	Chalkidiki	40.32′	23.25′	200	− 7.2	− 47.2
9	N.Triglia–N.Tenedos	Chalkidiki	40.34′	23.24′	250	− 7.3	− 48.1
10	Cave Petralona	Chalkidiki	40.36′	23.15′	900	− 9.5	− 62.1
11	Cave Petralona	Chalkidiki	40.36′	23.15′	900	− 9.7	− 66.7
12	Polygyros	Chalkidiki	40.37′	23.44′	800	− 9.2	− 61.8
13	Polygyros	Chalkidiki	40.37′	23.44′	800	− 9.2	− 58.9
14	Aridaia	Pela	40.97′	22.06′	1,100	− 9.6	− 68.5
15	Aridaia	Pela	40.97′	22.06′	1,100	− 9.7	− 67.9
16	Edessa	Pela	40.80′	22.10′	210	− 6.8	− 51.9
17	Edessa	Pela	40.79′	22.01′	330	− 7.5	− 53.2
18	Edessa	Pela	38.27′	23.63′	100	− 6.2	
19	Drossia (bottled)	Pela	40.80′	21.87′	1,000	− 9.1	− 62.0
20	Edessa	Pela	40.81′	22.01′	450	− 8.6	
21	Edessa	Pela	40.47′	21.52′	1,100	− 9.5	
22	Naoussa	Imathia	40.60′	22.04′	950	− 9.5	− 64.4
23	Naoussa	Imathia	40.61′	22.09′	950	− 9.3	− 64.0
**Central Greece**							
24	Magoula	Attiki	38.07′	23.52′		− 8.1	− 50.2
25	Magoula	Attiki	38.07′	23.52′		− 8.5	− 53.0
26	Pikermi	Attiki	38.00′	23.94′		− 7.2	− 42.5
27	Agia Marina	Attiki	37.74′	23.53′		− 5.8	− 36.3
28	Kalamos	Attiki	38.28′	23.86′		− 7.5	− 44.4
29	Kalamos	Attiki	38.28′	23.86′		− 7.3	− 43.1
30	Kalamos	Attiki	38.28′	23.86′		− 7.0	− 42.5
31	Monastiraki Vonitsas	Aitoloakarnania	38.84′	20.96′		− 7.9	− 55.9
32	Skasmeni	Fthiotida	38.82′	22.35′		− 9.0	− 60.0
33	Thermopyles	Fthiotida	38.79′	22.54′		− 7.3	− 55.5
34	A. Kostantinos	Fthiotida	38.75′	22.85′		− 6.3	− 42.1
35	Ioli Moschochori, (bottled)	Fthiotida			1,100	− 9.2	− 62.0
36	Evdoro (bottled)	Fthiotida	38.52′	22.14′		− 9.8	− 68.0
37	Velouchi kefalovriso, Ag. Triada	Fthiotida	38.97′	21.82′	1,800	− 9.9	− 67.0
38	Velouchi	Fthiotida	38.97′	21.79′	1,700	− 9.8	
39	Damasta monastiri 1	Fthiotida	38.78′	22.47′	750	− 7.8	− 50.1
40	Damasta monastiri 2	Fthiotida	38.78′	22.48	650	− 7.1	− 49.0
41	Anthili	Fthiotida	38.83′	22.47′		− 8.8	− 59.1
42	Anthili	Fthiotida	38.83′	22.47′		− 8.3	− 56.2
43	Anthili	Fthiotida	38.83′	22.47′		− 9.1	− 60.1
44	Platystomo	Fthiotida	38.96′	22.11′		− 9.7	− 59.8
45	Ypati Neochori	Fthiotida	38.80′	22.20′	1,100	− 9.2	− 65.1
46	Ypati kastanias	Fthiotida	38.85′	22.21′	1,050	− 8.7	− 54.9
47	korpi Monastiraki Vonitsa, (bottled)	Fthiotida	38.85′	20.94′	800	− 7.6	
48	Thermopyles	Boeotia	38.79′	22.51′	280	− 7.8	− 48.6
49	Agia Marina	Boeotia	38.56′	22.70′	1,100	− 9.3	− 57.1
50	Knimis spring	Boeotia	38.76′	22.74′	335	− 7.7	− 47.6
51	Monastery	Boeotia	38.78′	22.75′	277	− 7.7	− 49.3
52	Kamena Vourla	Boeotia	38.77′	22.79′	400	− 8.1	− 50.3
53	Orchomenos	Boeotia	38.49′	22.97′	800	− 8.5	− 53.6
54	Aliartos	Boeotia	38.37′	23.10′	600	− 7.8	− 48.7
55	Kalamos	Boeotia	38.16′	23.72′	1,000	− 9.0	− 55.0
56	Kalamos	Boeotia	38.17′	23.73′	1,160	− 8.9	− 54.0
57	Kalamos	Boeotia	38.14′	23.74′	550	− 7.8	− 45.0
58	Kalamos	Boeotia	38.18′	23.79′	590	− 8.1	− 53.0
**S. Greece (CRETE)**							
59	Psiloritis, Zaros, (bottled)	Irakleio	35.13′	24.90′	410	− 7.9	− 52.0
60	A. Mironas	Iraklio	35.23′	25.03′	1,200	− 9.6	− 59.0
61	Sarchos	Iraklio	35.22′	25.00′		− 8.5	− 51.3
62	Kroussonas	Iraklio	35.23′	24.98′	690	− 8.4	− 52.5
63	Archanes	Iraklio	35.23′	25.16′	800	− 9.2	− 58.3
64	Sterna	Irakleio	35.00′	25.09′	400	− 7.8	− 52.1
65	Amari	Irakleio	35.22′	25.65′	350	− 7.0	− 49.1
66	Irakleio	Irakleio	35.32′	25.16′		− 6.7	− 36.1
67	Nera Critis, (bottled)	Chania	35.47′	23.97′	800	− 8.7	− 51.0
68	Therisso	Chania	35.40′	23.98′	800	− 8.9	− 52.0
69	Chania	Chania	35.51′	24.02′		− 8.7	− 51.9
70	Spili	Rethymno	35.22′	24.54′	1,000	− 8.9	− 52.3
71	Stylos	Rethymno	35.43′	24.12′	600	− 8.2	− 50.1
72	Kourtalioti	Rethymno	35.20′	24.47′	300	− 6.2	− 35.1
73	Argyroupoli	Rethymno	35.28′	24.33′	250	− 5.9	− 33.4
74	Megali Vrisi	Iraklio	35.13′	25.02′	600	− 6.9	− 31.5
75	Plati	Iraklio	35.16′	25.44′	880	− 8.4	− 50.0

The human teeth samples (second maxillary molars) were collected at numerous dentist practices from Greece, in a period of 5–6 years and prior 2017, by the dentists, as part of routine treatments and were considered as waste for disposal. The patients were informed by the dentists that their teeth would be used for scientific studies and they gave their verbal consent. They were asked to answer a set of questions in order to document the samples. No record of the identity of the donors was kept but only relevant information as the area of their residence, their gender, their travel and their drinking habits during the first 20 years of their life. From these samples sixteen (16) were selected that match the areas of the study and from individuals that seldom traveled during their early life while systematically consuming tap water (tap water in Greece originates mostly from spring waters). These criteria were set in order the tooth enamel bioapatite of the selected samples to reflect the spring water composition of their area of origin. For these areas, tap water samples were also isotopically measured (^18^Ow vs SMOW). For the additional (14) teeth samples there was no conclusive information on the consumed water during the early years of the donors and were used as a case study of the methodology. In total, thirty teeth samples were isotopically analyzed (tooth enamel bioapatite carbon and oxygen versus PDB) from the areas of N. Greece (Pella, Chalkidiki, Imathia), of Central Greece (Attiki, Aitoloakarnania, Boeotia, Fthiotida) and from S.Greece (Crete).

The archeological samples from Roman Edessa and Medieval Thebes were human teeth (16 samples from Edessa and 11 from Thebes) and are from the same collection as described in our previous work^16,31^. The tooth enamel bioapatite carbon and oxygen isotopes versus PDB for all the samples was measured. The samples were not affected by the burial conditions. All samples produced acceptable atomic C/N ratios, (between 2.9 and 3.6)^50^.

The teeth samples were cleaned and powdered before analyzing as described in Martin et al.^51^. The samples reacted with ortho-phosphoric acid (99%) at 72 °C, to produce CO_2_ (GasBench II device) and were measured with a continuous flow mass spectrometer. The isotopic ratios of carbon (δ^13^C) and oxygen (δ^18^O) for tooth enamel bioapatite were measured versus PDB (marine carbonate).

The isotopic ratios of oxygen (δ^18^Ow) and hydrogen (δ^2^H) for the water samples were measured versus SMOW using the CO_2_–H_2_–water equilibration method according to the protocol described by Hilkert and Avak and Duhr and Hilker^52,53^.

The results are given in δ-notation (parts per mille—‰):

$$\delta = \left( {{\text{Rsample}}/{\text{Rstandard}} - {1}} \right)$$

where, R_sample_ and R_standard_ refer to ^2^H/^1^H or ^18^O/^16^O and ^13^C/^12^C ratios of sample and standard respectively. Blanks and duplicate samples were employed to evaluate analytical bias and precision. Analysis of blank samples did not show any inherent bias. The precision for δ^18^O, δ^13^C was ± 0.2 ‰ and for δ^2^H ± 1‰.

Isotopic analyses were conducted at the Unit of Stable Isotopes, Institute of Nanoscience and Nanotechnology, N.C.S.R. “Demokritos” on a continuous flow Finnigan DELTA V plus equipped with Gasbench device (Thermo Electron Corporation, Bremen, Germany) stable isotope mass spectrometer.

The isoscape (Fig. 2) was constructed by gridded isotopic data sets with a resolution of 30″ × 30″ (approximately 1 km × 1 km) according to the methodology as described in Bowen et al.^54^ and as implemented in Lykoudis et al. 2007 and Dotsika et al. 2010, 2018 ^10,11,55^. The GTOPO30 data set (maintained by the United States Geological Survey^56^) was used.

## Results

### Stable isotope of spring waters

**Figure 1:**
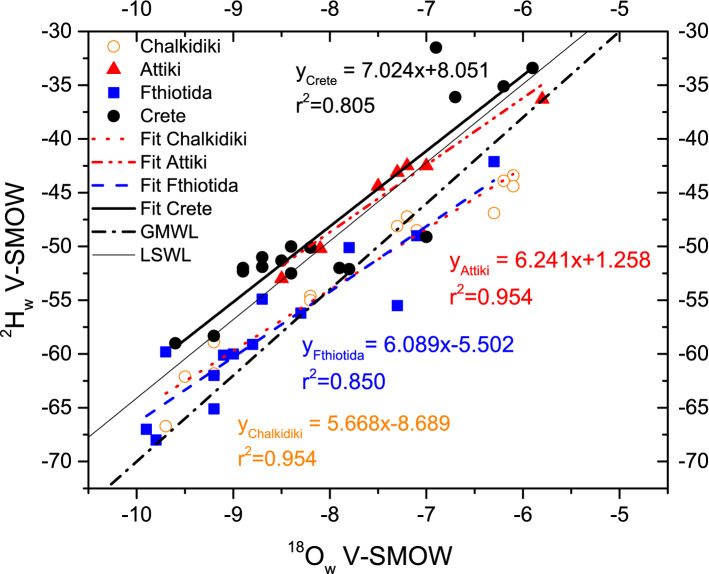
^18^O_w_ versus ^2^H_w_ for North (Chalkidiki), Central (Attiki and Fthiotoda) and South (Crete) Greece.

In the present study, more than 70 springs water were analyzed from the exactly same area of residency of the human donors of the teeth samples. The measured stable isotope ratios of spring water samples from North Greece (Chalkidiki) range between − 9.7‰ to − 6.1‰ for δ^18^O and from − 66.7‰ to − 43.4‰ for δ^2^H. The isotopic variation for Central Greece (Attiki and Fhthiotida) ranges from − 8.5‰ to − 5.8‰ for δ^18^O and from − 53.0‰ to − 36.3‰ for δ^2^H for Attiki, while for Fhtiotida from − 9.9‰to − 6.3‰ for δ^18^O and from − 68.0‰ to − 42.1‰ for δ^2^H. The isotopic variation for South Greece (Crete) ranges from − 9.6‰ to − 5.9‰ for δ^18^O and from − 59.0‰ to − 31.5‰ for δ^2^H. This observed isotopic variability reflects altitude, continental and latitude effects. Isotopic data of all spring waters are shown in Fig. 1.

The hydrogen and oxygen isotope ratios of spring water samples lie within a range of values typical of meteoric waters. The samples group near the global and Greek meteoric water line. These observations imply that the isotopic values of the spring waters might reflect a relatively unaltered meteoric water signature. Also, the isotope spring line from Chalkidiki (δ^2^H = 5.69δ^18^O − 8.69), Fthiotida (δ^2^H = − 6.09δ^18^O − 5.50), Attiki (δ^2^H = 6.24δ^18^O + 1.26), and Crete (δ^2^H = 7.02δ^18^O + 8.05) are shown in Fig. 1 and are in accordance to our previous works^10,11^. The observed decrease in spring waters slope in relation to the isotope ratios of Greek precipitation^10^ from South to North, show that these waters are affected by evaporation processes. Possible cause for this enrichment is the partial evaporation of water before the infiltration, the infiltration of recycled irrigation water, and the evaporation of soil water. Furthermore, the D-excess values (intercept of the fits to y-axis) progressively become more negative from South to North. Especially the negative values in Fthiotida and Chalkidiki indicate intense evaporation of the raindrops beneath the cloud base at high air temperatures^57^. This mechanism is probably balanced out in the case of Crete and in less extent for Attiki the due to intense evaporation of seawater in conditions of moisture deficit^58,59^.

**Figure 2:**
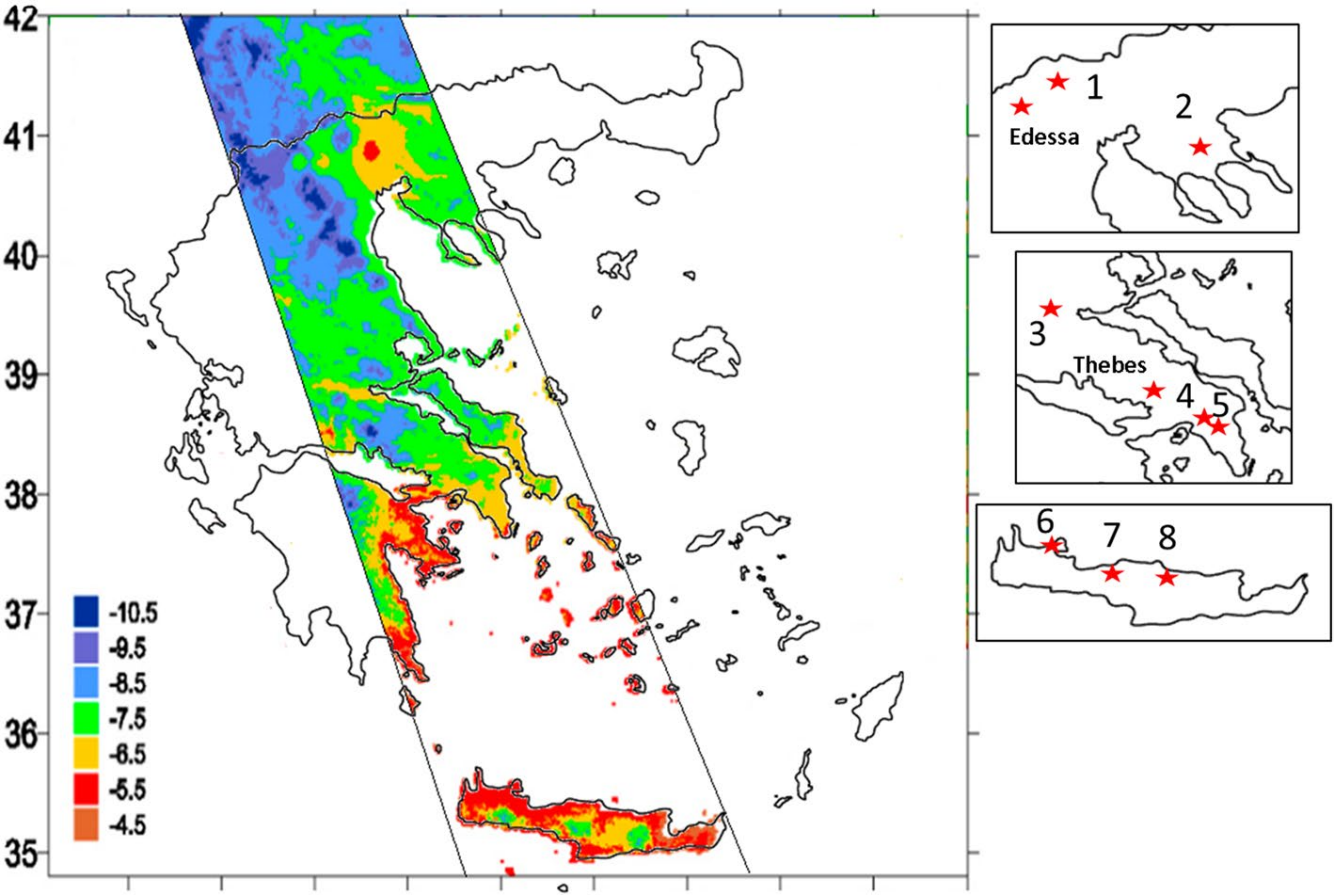
Spatial distribution of spring waters δ^18^O for North Greece (1. Pella-Aridaia, 2. Chalkidiki), Central Greece (3. Fthiotida, 4.Galatsi, 5. Ag. Paraskevi) and South Greece (6. Chania, 7. Rethimno, 8. Irakleio), Roman Edessa and Medieval Thebes. The figure was created according to methodology of Bowen et al.^54^ and the GTOPO30 data set (maintained by the United States Geological Survey^56^) was used.

Oxygen isotope composition of spring waters from Chalkidiki, Attiki, Fthiotida and Crete was used to improve the spatial variability of the isoscape map published from our previous work (Dotsika et al.)^10,11^ according to the methodology of Bowen et. al.^54^. In order to achieve the highest possible resolution the GTOPO30 data set maintained by the United States Geological Survey (USGS, 2008), was used. In Fig. 2, a revised map of the spring waters for the areas denoted in Table 1 is presented that is slightly different from the map in our previous work^11^, especially near the sampling regions (see inserts of Fig. 2) as more data were implemented (Table 1).

**Figure 3:**
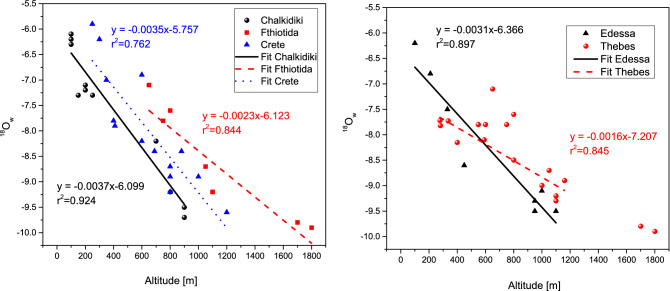
Oxygen isotope values of selected spring waters versus Altitude Gradient. (**a**) from Chalkidiki, Fthiotida and Crete, (**b**) from Edessa (Boeotia region) and Thebes (Pella region).

In Fig. 3a the Oxygen isotope values of selected spring waters from Fthiotida Chalkidiki and Crete are presented, with known altitude of the supply reservoir of the springs (data from Table 1). The isotopic gradients for the studied areas are estimated by regression lines (δ^18^O = a* Altitude + b), which were fitted on the available data from each area. These local-specific gradients for Chalkidiki, Fthiotida and Crete are: δ^18^Ο = − 0.0037*Altitude − 6.01, δ^18^O = − 0.0035* Altitude − 5.76 and δ^18^O = − 0.0023* Altitude − 6.21 respectively.

These equations enable the approximate determination of the mean altitude of the human’s (and animals) habitats through the variation pattern of the isotopic composition of their teeth. Practically every 100 m of altitude difference the δ^18^Ow values vary (decrease as altitude rises) as 0.37‰, 0.35‰ and 0.23‰ for North, Central and South Greece respectively. This correlation of the δ^18^Ο_w_ values versus altitude can potentially provide valuable explanations of the measured δ^18^O_en_ range of unknown samples both contemporary and archeological. At that end this correlation is implemented in two case studies (one with contemporary samples and one with archeological samples from Medieval Thebes and Roman Edessa. Especially for the archeological case study, the Oxygen isotope values versus altitude for Thebes and Edessa were calculated from the values of Boeotia and Pella, respectively (Fig. 3b, Table 1). For Thebes and Edessa the local-specific gradient of oxygen isotope value versus altitude (δ^18^O = a* Altitude + b) are: δ^18^O = − 0.0016* Altitude − 7.21 and δ^18^O = − 0.0031* Altitude − 6.37, respectively.

### Human data, δ^18^Oen_C_, for forensic studies

Bioapatite has a general form of Ca_10_(PO_4_,CO_3_)_6_(OH,CO_3_)_2_ and therein the oxygen isotope can be determined either by measuring the structural carbonate (CO_3_^2–^) or the phosphate (PO_4_^3–^). Both these ions oxygen isotope composition can be related to body water and finally to the consumed water. In tooth enamel bioapatite, phosphate oxygen ion is predominant than structural carbonate and the P–O bond is stronger than the C–O bond, thus consisting the phosphate ion more resistant to alterations than the structural carbonate^60^. Nevertheless, structural carbonate is easier and quicker to measure and as was demonstrated in Chenery et al.^50^, the ^18^O_C_ values as derived from structural carbonate in human tooth enamel bioapatite (for the last 5,000 years) can be considered accurate and precise.

The isotopic analysis of ^18^Oen_C_ of 30 tooth enamel bioapatite originating from three different areas of Greece: North Greece (Pella-Aridaia, Chalkidiki-Ierisos), Central Greece (Fthiotida-Lamia, Velouchi and Attiki-Athens, Galatsi, Ag. Paraskevi) and South Greece (Crete-Rethimno, Irakleio) are given in Table 2. Among these samples, special “marker” samples (16 samples in bold in Table 2) were selected, for which the following are exactly known: the kind of water they consumed and where they lived and that these individuals did not travel for long periods during the early years of their life. For these samples isotopic analysis (δ^18^Ow VSMOW) of the tap water from the respectively regions that these donors consumed (Table 2—8th column), was conducted.

**Table 2:** Oxygen isotopic composition of human teeth and of spring water samples from the same regions.

	Sample	District	Town	^13^Cen V-PDB	^18^Oen_C_ V-PDB	^18^Oen_C_ V-SMOW	^18^Ow V-SMOW measured
**N.Greece**							
**1**	**HM1AR**	Pella	Aridaia	− 12.4	− 8.4	22.7	− 9.7
**2**	**HM2AR**	Pella	Aridaia	− 12.8	− 8.1	23.0	− 9.7
**3**	**HM1CH**	Chalkidiki	Ierisos	− 11.2	− 5.3	25.8	− 6.5
**4**	**HM2CH**	Chalkidiki	Ierisos	− 11.0	− 5.3	25.8	− 6.5
**5**	**HM3CH**	Chalkidiki	Ierisos	− 11.4	− 5.6	25.5	− 6.5
**Central Greece**							
**6**	**HM2FTl**	Fthiotida	Lamia	− 11.0	− 5.5	25.6	− 8.3
**7**	**HM4FTl**	Fthiotida	Lamia	− 12.2	− 6.0	25.1	− 8.3
**8**	**HM6FTv**	Fthiotida	Velouchi	− 13.4	− 7.8	23.3	− 9.5
**9**	**HM7FTv**	Fthiotida	Velouchi	− 14.5	− 8.3	22.8	− 9.5
**10**	**HM1A.P**	Attiki	A.Paraskevi	− 11.5	− 6.0	25.1	− 6.9
**11**	**HM2A.P**	Attiki	A.Paraskevi	− 7.5	− 5.8	25.3	− 6.9
**12**	**HM5A.P**	Attiki	A.Paraskevi	− 7.3	− 5.4	25.7	− 6.9
13	HM1FT	Fthiotida	Lamia	− 9.0	− 3.9	27.2	
14	HM3FT	Fthiotida	Lamia	− 10.1	− 4.9	26.2	
15	HM5FT	Fthiotida	Lamia	− 13.0	− 6.6	24.5	
16	HM7FT	Fthiotida	Fthiotida	− 11.0	− 5.3	25.8	
17	HM1AT	Attiki	Athens	− 9.8	− 3.8	27.3	
18	HM3A.P	Attiki	A.Paraskevi	− 12.2	− 2.1	29.0	
19	HM4A.P	Attiki	A.Paraskevi	− 12.8	− 7.6	23.5	
20	HM1GA	Attiki	Galatzi	− 11.0	− 5.5	25.6	
**S.Greece**							
**21**	**HM1CRr**	Crete	Rethymno	− 11.2	− 3.6	27.5	− 4.5
**22**	**HM4CRr**	Crete	Rethymno	− 7.1	− 2.6	28.5	− 4.5
**23**	**HM6CRi**	Crete	Iracleio	− 12.8	− 6.6	24.5	− 7.8
**24**	**HM8CRi**	Crete	Iracleio	− 13.1	− 6.9	24.2	− 7.8
25	HM9CRi	Crete	Iracleio	− 11.5	− 6.6	24.5	
26	HM10CRi	Crete	Iracleio	− 10.5	− 7.6	23.5	
27	HM3CRr	Crete	Rethymno	− 11.5	− 5.7	25.4	
28	HM5CRi	Crete	Iracleio	− 11.2	− 5.6	25.5	
29	HM7CRi	Crete	Iracleio	− 11.5	− 5.8	25.3	
30	HM2CRr	Crete	Rethymno	− 8.4	− 1.8	29.3	

North Greece: *AR* Aridaia, *CH* Chalkidiki; Central Greece: *AT* Athens, *A.P* Agia Paraskevi, *GA* Galatzi, *FT* Fthiotida, *FTv* Velouchi, *FTl* Lamia; South Greece: *CRr* Crete-Rethymno; *CRi* Crete-Iracleio.

Considering that the major factor that affects the oxygen isotopic composition of human teeth is the consumed water, the observed variation of the isotopic composition of contemporary teeth is modulated by the climatic conditions of each individual’s place of residence: the apparent differences of the oxygen isotopic composition of samples can be translated mainly into environmental conditions.

**Figure 4:**
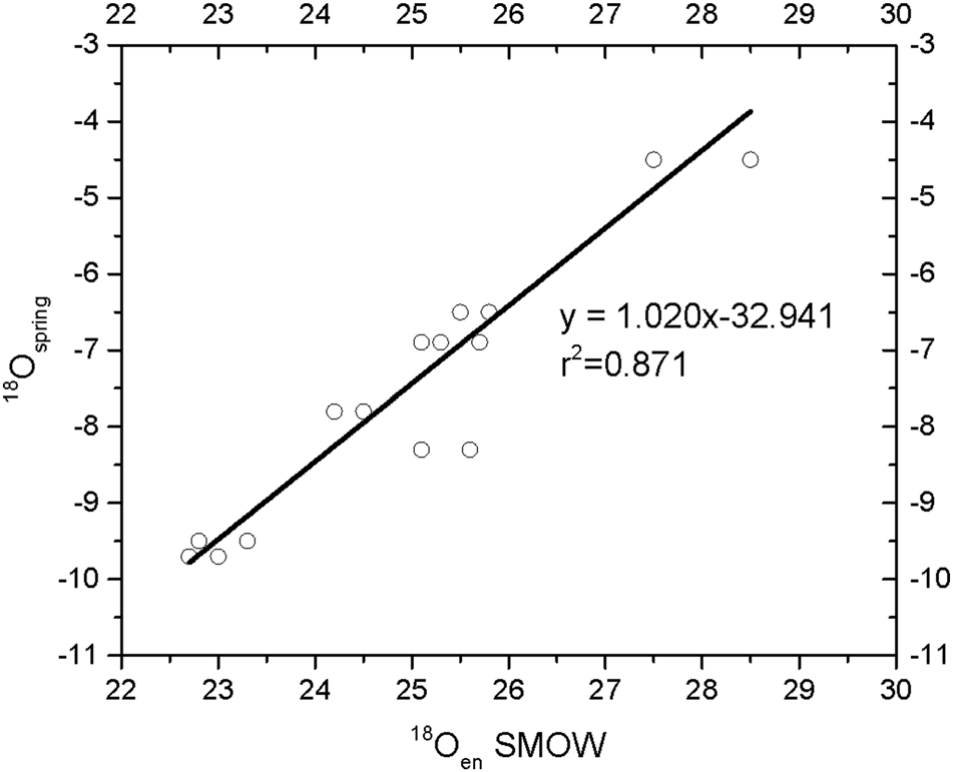
Spring water oxygen (ingested water), δ^18^O_w_ *versus* tooth enamel bioapatite oxygen, δ^18^Oen_C_.

The δ^18^Oen_C_ values of tooth enamel bioapatite were converted from VPDB to VSMOW according to the equation proposed by^61^ and used in^42^: ***δ***^18^O_V–SMOW_ = 1.03091 ***δ***^18^O_PDB_ + 30.91. Using the values of δ^18^Ow and δ^18^Oen_C_ (Table 2, Fig. 4) that correspond to the “marker” samples, an equation between δ^18^O_en_ and δ^18^Ow for the modern bone apatite is formed:

1 $$\delta^{{{18}}} {\text{Ow }} = {1}.0{2}0\delta^{{{18}}} {\text{Oen}}_{{\text{C}}} - {32}.{941 }\left( {{\text{r}}^{{2}} = 0.{87},{\text{ n}} = {16}} \right)$$

This formula, reversed in order to represent the dependence of human teeth δ^18^Oen_C_ on δ^18^Ow, is quite similar to the δ^18^Oen_P_ obtained by tooth and bone enamel in the literature:

Longinelli. 1984 ^41^,

2 $$\delta^{{{18}}} {\text{O}}_{{\text{W}}} = {1}.{53}*\delta^{{{18}}} {\text{Oen}}_{{\text{P}}} - {34}.{3},\left( {{\text{r}}^{{2}} = 0.{97},{\text{ n}} = {1}0} \right)$$

Luz et al., 1984 ^44^,

3 $$\delta^{{{18}}} {\text{O}}_{{\text{W}}} = {1}.{19}*\delta^{{{18}}} {\text{Oen}}_{{\text{P}}} - {27}.{42}, \, \left( {{\text{r}}^{{2}} = 0.{95},{\text{ n}} = {6}} \right)$$

Levinson et al. 1987^43^,

4 $$\delta^{{{18}}} {\text{O}}_{{\text{W}}} = {1}.{93}*\delta^{{{18}}} {\text{Oen}}_{{\text{P}}} - {38}.{51}, \, \left( {{\text{r}}^{{2}} = 0.{92},{\text{ n}} = {4}0} \right)$$

Daux et al., 2008^40^,

5 $$\delta^{{{18}}} {\text{O}}_{{\text{W}}} = {1}.{73}*\delta^{{{18}}} {\text{Oen}}_{{\text{P}}} - {37}.{2}, \, \left( {{\text{r}}^{{2}} = 0.{87},{\text{ n}} = {38}} \right)$$

The ratio relating δ^18^Oen and δ^18^Ow is equal to 1.02 (Fig. 4). This equation incorporates only the effect of drinking water on the isotopic composition of body water. Similar relationships for herbivores (between δ^18^Oen_C_ and δ^18^Ow) have a slope less than 1^41,42,62^, whereas for carnivores have a larger than 1. So it is reasonable for an omnivore species, like humans, to have a slope value equal or little larger than 1.

From the measured δ^18^Oen_C_ values of the marker samples (Table 2-last column) and with the use of the Eq. (1) the δ^18^Ow_C_ values of the consumed water (Table 3-column 6) were calculated. Similarly, the δ^18^Ow_C_ with all the data sets of the literature (Eqs. 2–5), were also calculated. For this calculation the measured carbonate tooth enamel bioapatite ^18^Oen_C_ values were converted to phosphate values ^18^Oen_P_ according to Pellegrini et al.^63^ and with the equation δ^18^O_P_ = (1.00867 ± 0.00077)*δ^18^O_C_-(8.8 ± 0.8‰) and the results are given at column 4 of Table 3. For comparison, the mean relative difference was also calculated:

$${\text{R}}_{{{\text{error}}}} = {\text{ abs}}[(\delta^{{{18}}} {\text{Ow}}_{{{\text{measured}}}} - \delta^{{{18}}} {\text{Ow}}_{{{\text{calculated}}}} )*{1}00)/\delta^{{{18}}} {\text{Ow}}_{{{\text{measured}}}} ]$$

of the δ^18^Οw_calculated_ for the marker samples and the measured δ^18^Οw_measured_ values (Table 3 column 5) and was found equal to 5.44%. The mean differences for the four equations of the literature were: 18.180% for Levinson, 30.89% for Longinelli, 13.70% for Luz and 28.22% for Daux. Equation 1, yielded the less mean difference (5.44%) and that reflects—in my opinion- the local nature of the data set of this study, compared to the data sets of the literature which span in vast geographic regions^40,41,43,44^.

**Table 3:** Calculated oxygen isotopic composition of water consumed according to this work equation and to the available equations of the literature.

	Sample	^18^Oen_C_ measured	^18^Oen_P_ calculated	^18^Ow measured	^18^Ow calculated					Relative difference: Abs[(^18^Ow_M_-^18^Ow_c_)*100/^18^Ow_M_]				
					THIS Study	Levinson	Longinelli	Luz	Daux	This study	Levinson	Longinelli	Luz	Daux
**N.Greece**														
**1**	**HM1AR**	22.7	13.7	− 9.7	− 9.8	− 12.1	− 13.4	− 11.1	− 13.5	1.49	24.80	37.82	14.84	39.50
**2**	**HM2AR**	23.0	14.0	− 9.7	− 9.5	− 11.5	− 12.9	− 10.8	− 13.0	4.59	18.88	33.12	11.19	34.20
**3**	**HM1CH**	25.8	16.8	− 6.5	− 6.6	− 6.2	− 8.7	− 7.5	− 8.2	0.79	5.01	33.32	15.11	26.39
**4**	**HM2CH**	25.8	16.8	− 6.5	− 6.6	− 6.2	− 8.7	− 7.5	− 8.2	0.79	5.01	33.32	15.11	26.39
**5**	**HM3CH**	25.5	16.5	− 6.5	− 6.9	− 6.7	− 9.1	− 7.8	− 8.7	3.84	3.82	40.32	20.56	34.30
**Central Greece**														
**6**	**HM2FTl**	25.6	16.6	− 8.3	− 6.8	− 6.6	− 9.0	− 7.7	− 8.6	19.89	21.00	8.06	7.01	3.11
**7**	**HM4FTl**	25.1	16.1	− 8.3	− 7.3	− 7.5	− 9.7	− 8.3	− 9.4	13.85	9.47	17.20	0.10	13.44
**8**	**HM6FTv**	23.3	14.3	− 9.5	− 9.2	− 11.0	− 12.5	− 10.4	− 12.5	5.74	15.34	31.14	9.81	31.61
**9**	**HM7FTv**	22.8	13.8	− 9.5	− 9.7	− 11.9	− 13.2	− 11.0	− 13.4	0.47	25.41	39.12	16.02	40.64
**10**	**HM1A.P**	25.1	16.1	− 6.9	− 7.3	− 7.5	− 9.7	− 8.3	− 9.4	3.63	8.89	40.98	20.41	36.46
**11**	**HM2A.P**	25.3	16.3	− 6.9	− 7.1	− 7.1	− 9.4	− 8.1	− 9.1	0.72	3.35	36.59	16.99	31.49
**12**	**HM5A.P**	25.7	16.7	− 6.9	− 6.7	− 6.4	− 8.8	− 7.6	− 8.4	5.09	7.74	27.79	10.15	21.55
13	HM1FT	27.2	18.1		− **5.2**	− 3.5	− 6.5	− 5.8	− 5.8					
14	HM3FT	26.2	17.2		− **6.2**	− 5.4	− 8.1	− 7.0	− 7.5					
15	HM5FT	24.5	15.5		− **7.9**	− 8.7	− 10.6	− 9.0	− 10.4					
16	HM7FT	25.8	16.8		− **6.6**	− 6.2	− 8.7	− 7.5	− 8.2					
17	HM1AT	27.3	18.2		− **5.0**	− 3.3	− 6.4	− 5.7	− 5.6					
18	HM3A.P	29.0	19.9		− **3.4**	− 0.1	− 3.8	− 3.7	− 2.7					
19	HM4A.P	23.5	14.5		− **8.9**	− 10.6	− 12.2	− 10.2	− 12.2					
20	HM1GA	25.6	16.6		− **6.8**	− 6.6	− 9.0	− 7.7	− 8.6					
**S. Greece**														
**21**	**HM1CRr**	27.5	18.4	− 4.5	− 4.9	− 2.9	− 6.1	− 5.5	− 5.3	5.44	35.08	35.28	21.71	17.77
**22**	**HM4CRr**	28.5	19.4	− 4.5	− 3.9	− 1.0	− 4.6	− 4.3	− 3.6	16.83	77.59	1.57	4.51	20.35
**23**	**HM6CRi**	24.5	15.5	− 7.8	− 7.9	− 8.7	− 10.6	− 9.0	− 10.4	0.62	11.05	36.38	15.59	33.91
**24**	**HM8CRi**	24.2	15.2	− 7.8	− 8.2	− 9.2	− 11.1	− 9.4	− 11.0	3.24	18.41	42.22	20.13	40.50
25	HM9CRi	24.5	15.5		− **7.9**	− 8.7	− 10.6	− 9.0	− 10.4					
26	HM10CRi	23.5	14.5		− **8.9**	− 10.6	− 12.2	− 10.2	− 12.2					
27	HM3CRr	25.4	16.4		− **7.0**	− 6.9	− 9.3	− 8.0	− 8.9					
28	HM5CRi	25.5	16.5		− **6.9**	− 6.7	− 9.1	− 7.8	− 8.7					
29	HM7CRi	25.3	16.3		− **7.1**	− 7.1	− 9.4	− 8.1	− 9.1					
30	HM2CRr	29.3	20.2		− **3.1**	0.5	− 3.4	− 3.4	− 2.2					
	**Mean error (%)**									5.44	18.18	30.89	13.70	28.22

The calculated values of the consumed water δ^18^Οw_C_ (Table 3, column 6) coincide with the δ^18^Οw values of spring waters (Figs. 2 and 3) with the exception of one sample for S. Greece. This sample (HM4CRr) with value δ^18^Οw_C_ = − 3.9‰ is outside the δ^18^Ο_w_ value range of the region (Rethimno-Crete) which is from − 4.5‰ to − 8.0‰.

## Discussion

### Case study 1: modern teeth of unknown origin compared to the area of collection

For the 14 samples with unknown water consumption Eq. (1) was used in order to predict potential origins. The results (^18^Ow_calculated_) are presented in Table 3-column 6 (in bold). The uncertainty of the calculation is assumed 5.5% as derived from the relative difference of the 16 known samples in Table 3. In order to determine the origin of these samples, the calculated δ^18^Ow_C_ values of the consumed water were compared to the δ^18^Οw values of the spring waters of the corresponding area from where the samples were collected (Fig. 2).

For the samples collected in Central Greece, it is concluded that four of them (HM3FT, HM5FT, HM7FT and HM1GA) with δ^18^Οw_C_ values ranging from − 6.2‰ to − 7.9‰ have values in accordance with the area they were collected suggesting that the individuals consumed water from Fthiotida (− 6.0‰ to − 8.5‰) and the East Attiki (− 7.8‰ to − 6.2‰).The samples HM1AT (Athens), HM3A.P and HM4A.P (Ag. Paraskevi) with calculated δ^18^Ο_WC_ values of − 5.0‰, − 3.4‰ and − 8.9‰ respectively fall outside of the East Attika values range for ^18^O_w_. The same applies to the sample HM1FT from Fthiotida with calculated δ^18^Οw_C_ value of − 5.2‰. The calculated values of these samples suggest that these individuals can be considered ‘non-local’ to Fthiotida, Athens and Ag. Paraskevi in the sense of consuming water not belonging to the local reservoirs. Please note that Attiki region water supply originates from various sources and is collected to several aqueducts for further distribution, thus, as a result of the mixing, the potential altitude difference of the various water sources cannot be considered in order to explain the δ^18^Οw_C_ values of the samples HM1AT (Athens), HM3A.P and HM4A.P (Ag. Paraskevi). Furthermore, Fthiotida region water resources in general originates from high altitudes since the measured ^18^Ow values range from − 6.0‰ to − 8.5‰ vs SMOW. The calculated δ^18^Ow_C_ values for the HM1FT sample (− 5.2‰) indicate a water source of considerably lower altitude compared to the available sources of the region. For the samples collected in South Greece, all the samples have δ^18^Οw_C_ values (ranging from − 6.9‰ to − 7.9‰) in accordance with the area they were collected suggesting that the individuals consumed water belonging to the local reservoirs with the exception of samples HM10Cri and HM2CRr. The sample HM10Cri yielded the most negative value (− 8.9‰). This value can be explained by the consumption of waters from the mountain of Crete (“Zaros” − 7.9‰, and “Nera Critis” − 8.7‰ for the δ^18^O) (Table 1). Contrary, the calculated δ^18^Οw_C_ value of sample HM2CRr (− 3.1‰) cannot be explained but only as originating from a ‘non-local’ donor or by other factors, i.e. drinking water not originating from the local spring; treatment of food (boiling water); consummation of drinks (like milk). Especially the last two produce enrichment in δ^18^O of water.

### Case study 2: Archaeological human data for paleomobility studies

The model is applied to paleomobility studies by analyzing the oxygen isotope values of tooth enamel bioapatite from Roman Edessa (N. Creece) and Medieval Thebes (Central Greece-Boeotia). In Edessa, the enamel oxygen sotope values range between − 5.7‰ to − 9.2‰ (V PDB), whereas in Thebes between − 3.0‰ to − 9.1‰ (V PDB), see Table 4. The mean oxygen enamel difference between the two sites is statistically significant: δ^18^Oen_C_ for Edessa is − 7.7 ‰; δ^18^Oen for Thebe is − 5.8 ‰ V PDB, which can be explained by the altitude and precipitation difference of the cities (see below).

**Table 4:** Archeological samples from Roman Edessa and Medieval Thebes. Isotopic measurements.

		Age	δ^13^Cen vs PDB	δ^18^Oen_C_ vs PDB	δ^18^Oen_C_ vs SMOW	δ^18^Ow_C_ vs SMOW Calculated
**EDESSA**						
E/T36NN	Male	20–35	− 11.8	− 5.7	25.4	− 7.5
E/T15	Male	36–50	− 9.9	− 6.9	24.2	− 8.7
E/T23	Male	36–50	− 11.7	− 7.7	23.4	− 9.5
E/T9	Male	20–35	− 12.2	− 7.3	23.8	− 9.1
E/T27B	Male	20–35	− 11.0	− 5.8	25.3	− 7.6
E/T4B	Female	36–50	− 10.5	− 8.7	22.4	− 10.5
E/T29	Female	20–35	− 12.2	− 8.8	22.3	− 10.6
E/T20BN	Female	20–35	− 12.6	− 8.0	23.1	− 9.8
E/T40B	Female	36–50	− 10.0	− 9.2	21.9	− 11.0
E/T35	Female	20–35	− 11.0	− 8.9	22.2	− 10.7
E/T32	Female	20–35	− 13.0	− 8.6	22.5	− 10.4
E/36NNA	Female	20–35	− 11.5	− 7.9	23.2	− 9.7
E/T27	Female	20–35	− 10.6	− 9.0	22.1	− 10.8
E/T25	Female	36–50	− 13.0	− 7.0	24.1	− 8.8
E/T26	Female	36–50	− 13.9	− 6.7	24.4	− 8.5
E/T36BN	Female	20–35	− 12.4	− 6.9	24.2	− 8.7
**Mean**				− 7.7	23.4	− 9.5
**THEBES**						
Th15	Male	20–35	− 13.5	− 9.5	21.6	− 11.3
Th20	Male	51 +	− 8.2	− 3.0	28.1	− 4.8
Th8	Male	20–35	− 7.5	− 5.0	26.1	− 6.8
Th9	Male	36–50	− 7.5	− 6.1	25.0	− 7.9
Th21	Male	36–50	− 6.5	− 9.1	22.0	− 10.9
Th13	Female	20–35	− 6.5	− 5.8	25.3	− 7.6
Th23	Female	20–35	− 7.5	− 5.0	26.1	− 6.8
Th12	Female	20–35	− 8.0	− 7.3	23.8	− 9.1
Th18	Female	20–35	− 3.4	− 3.7	27.4	− 5.5
Th3	Female	36–50	− 10.0	− 6.0	25.1	− 7.79
Th14	Female	36–50	− 6.0	− 3.3	27.8	− 5.09
**Mean**				− 5.8	25.3	− 7.6

In order to implement the methodology to archaeology, oxygen isotopic analysis was conducted to the water sources of different altitudes of the regions (^18^O_w_). Then these were correlated to climate data and the geographic spread of the ancient cities (since the population of an ancient city could have been water supplied from sources of different altitudes) in order to define the isotopic fingerprint of the areas. In overall, the climate of Greece has not changed considerably over the studied periods^13,48,49^.

**Figure 5:**
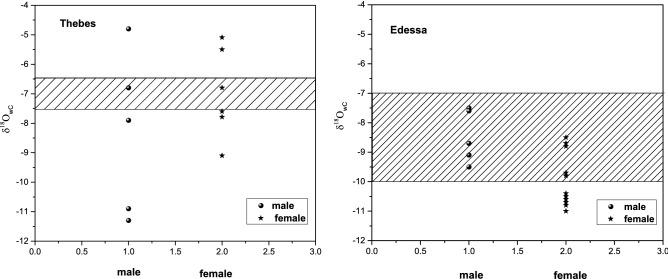
δ^18^Οw_C_ values of the consumed waters for the archeological samples of Medieval Thebes and Roman Edessa. The shaded areas are the δ^18^Ow ranges of spring waters of the area.

The annual precipitation rate is at 500–600 mm for Boeotia while the mean annual precipitation rate for Edessa is at 500 mm; however it exceeds 750 mm in regions of higher altitude (mountainous areas). The mean annual temperature for Boeotia is between 16–18 °C, while in Edessa is 12 °C. In addition, δ^18^O values for spring waters in Thebes range between − 6.5 to − 7.5‰^10,11^, while the oxygen isotopic signature for spring waters in Edessa varies from − 7 to − 10‰^10^. In Fig. 5 the ranges of the spring waters of Edessa and Thebes (the pattern filled rectangular) as well as the calculated consumed waters of the archeological samples, δ^18^Οw_C_, (Eq. 1) as well as their respective mean values, V SMOW, are presented. The uncertainty of the calculated consumed waters ^18^O_wc_ values is assumed 5.4% as derived from the relative difference of the 16 known samples in Table 3.

Based on the information presented above, more negative oxygen isotope values are expected in Edessa in relation to Thebes, as Edessa’s climate is colder and the Macedonian town presents a higher altitude (350 m) and a higher precipitation rate in relation to Thebes (200 m). In fact, oxygen enamel results seem to verify the expected pattern: the difference of δ^18^O of water, which is reflected to δ^18^O of teeth, is in accordance to what is expected from these differences on precipitation and altitude. Edessa is supplied by the water of mountain Voras (with mean altitude 1400 m) while Thebes from mountain Elikona (with mean altitude 600 m). Using the estimated gradient between δ^18^Ow and altitude for the two sites (− 0.16‰/100 m for Thebes and − 0.31‰/100 m for Edessa, Fig. 3b), the observed isotopic difference of 1.5–2‰ between the two sites is in accordance to a hypsometrical difference of approximately 800–1,000 m. If it was assumed that Thebes is 800 m higher in altitude (in order to match the water supply altitude of Edessa), the effect on the δ^18^Οw would be (− 0.16‰/100 m)*800 m which equals to 1.3‰. Subtracting this value to the mean value of Thebes (− 7.6‰) gives − 8.9‰, a value very close to the mean value of Edessa (− 9.5‰). Similarly, if it was assumed that Edessa is 800 m lower in altitude (in order to match the water supply altitude of Thebes), the effect on the δ^18^Οw would be (− 0.31‰/100 m)*800 m which equals to 2.5‰. Adding this value to the mean value of Edessa (− 9.5‰) gives − 7.0‰, a value very close to the mean value of Thebes (− 7.6‰).

By considering the δ^18^Οw values of each individual in Edessa or Thebes, the first observation is that the δ^18^Ow isotopic values ranges 2‰ for males and 2‰ for females of Edessa while for Thebes the range for males is 6‰ and for females 4‰. The second observation is that the majority of the individuals in Edessa have values within the ranges of the contemporary spring waters of the area (shaded area in Fig. 5) with the exception of six women. Contrary, the majority of the individuals in Thebes have values outside the contemporary spring waters of the area (shaded area in Fig. 5), both men and women.

Furthermore, bashed on the negative slopes of Fig. 3a,b, it is plausible to argue that the oxygen isotopic composition of waters from lower altitudes is enriched in ^18^O compared to waters of higher origin in a drainage basin. This effect could result to a difference in δ^18^O of tooth enamel biapatite from two individuals dwelling at different latitudes within that drainage basin. Following this reasoning it would seem that the six women of Edessa were dwelling in higher altitudes than the men.

A plausible assumption concerning the geographic distribution of the ancient local population is a minimum difference of altitude for a human’s habitat not higher or lower than 100 m (350 ± 100 m for Edessa and 200 ± 100 m for Thebes). This altitude difference can justify an isotopic difference of only 0.6‰ for Edessa and only 0.3‰ for Thebes (Fig. 3b) in the δ^18^O_w_. In order to justify the ^18^Ow isotopic values ranges (2‰ for males and 2‰ for females of Edessa and 6‰ for males and 4‰ for females of Thebes) the corresponding hypsometrical difference would be approximately 1,000 m for Edessa and 4000 m for Thebes (according to the slopes of Fig. 3b). This altitude difference is too high to correspond to different sources of drinking water originating from reservoirs of the local groundwater for Edessa and outside the height range of Greece for Thebes. These values could be affected by the different climatic conditions for individuals that lived in different areas and time periods during their childhood and the formation of their teeth. In fact, Edessa’s population covers a time period of 200 years, while Thebes of 100 years. Thus, it is quite possible that shifts between more open or forested habitats could have affected the human isotopic composition. Individuals that occupy open habitats ingest more positive δ^18^O in relation to individuals living in a cooler, moister forested habitat. Body size and metabolism can also influence the oxygen isotope composition of tooth enamel bioapatite. Large mammals that are obligate drinkers and tend to have lower metabolisms are more likely to track the δ^18^O values of drinking waters^41,44^. However the variability of 6‰ and 4‰ (males and females of Thebes) or even 2‰ and 2‰ (males and females of Edessa) in the ^18^O values is too great to be attributed in these climatic fluctuations.

Another parameter to consider is the consuming of liquids other than water and the effect to the δ^18^Οen values. Generally, about 70% of the water intake in human originates from consumed liquids while the rest 30% originates from food consumption^64^. In Table 5 some characteristic δ^18^Ο values of beverages are presented.

**Table 5:** Oxygen isotope values of beverages.

	Number of samples	Mean δ^18^Ο vs SMOW
Wine	300	3 ± 2‰
Fruit juices	50	2 ± 1‰
Milk	20	1 ± 1‰

If a simple mass equation is applied on the water intake of a human with, for example 60% water and 10% milk then:

$$\delta^{{{18}}} {\rm O}_{{{\text{en}}}} = 0.{6}\left( { - {8}\permille} \right) + 0.{1}\left( {{1}\permille} \right) + 0.{3}\left( {{\text{X}}\permille l} \right) = - {4}.{7}\permille + 0.{\text{3X}}\permille,$$

where X‰ is the mean Oxygen isotopic value for consumed food.

A variation on the above equation, for example 20% milk and 50% water will result to:

$$\delta^{{{18}}} {\rm O}_{{{\text{en}}}} = 0.{5}\left( { - {8}\permille} \right) + 0.{2}\left( {{1}\permille} \right) + 0.{3}\left( {{\text{X}}\permille} \right) = - {3}.{8}\permille + 0.{\text{3X}}\permille$$

Assuming the same X value for both cases almost 1‰ variation on the δ^18^Οen value is observed, by only replacing 10% of the consumed water with milk. In extreme cases that the water intake is reduced even further and replaced by milk or other beverages, even wine, we can get up to 2–2.5‰ difference (with 30% water and 40% milk).

Taking into account the above results it is plausible to speculate that the observed 2‰ range in the δ^18^Οw for Edessa (for males and females) can be justified considering the 0.6‰ variation due to altitude difference of the water reservoirs within the city and with the 1‰ variations due to different liquids intake of the individuals. Another observation on the six women with the negative δ^18^Οw values (outside the shaded area on Fig. 5) is that these women might be solely consuming water as their liquid intake (the above mass equations with 70% water and 30% food results to 1‰ more negative values). That might be an indication of the social status of these women—having no access to beverages other than water rather than dwelling in higher altitudes than that of the men. This conclusion is supported by the paleodiet analysis that was conducted to the same collection^15^, where no significant diet differences were detected to the Roman population of Edessa between males and females. In that study, females exhibit more negative values than males in all three carbon isotopic signatures, possibly suggesting the consumption of more terrestrial sources than men, while females present a higher caries rate than males. Nevertheless, this difference is not statistically significant, and thus could probably be attributed to biological and social factors.

Contrary, for the individuals of Thebes, the above results on the effect of introducing alternative beverages than water on the diet along with the 0.3‰ variation due to altitude difference of the water reservoirs within the city cannot justify the 6‰ and 4‰ range (for males and females, respectively) on the ^18^Ow values. Since enamel apatite fingerprint basically remains unaltered (i.e.^46,47^) it is quite possible that enamel values reflect more accurately the isotopic signals of the populations under study. This observed variability could also be attributed to the possible existence of several immigrants^31^. This conclusion is supported by the paleodiet analysis that was conducted to the same collection^16^ where a significant dietary diversity has been established confirming the historical sources (Medieval Thebes was ruled by the Franks (1,204–1,311), the Catalans (1,311–1,379), the Navarrese Company (1,379–c.1,388) and the Florentines (ca.1,388–1,460)). Dietary diversity could reflect class distinctions between the local people and visitors patronizing the area or could also be attributed to ethnic/cultural differences between the co-existing common people of the era (i.e. Greeks, Jews, Armenians and Albanians). In addition, this dietary diversity could be attributed to temporal changes due to historical conditions (the Catalan occupation was harsher in relation to the Frankish one, therefore resulting to different diet).

## Conclusions

A methodology for identify the geographic region from which an unidentified person originates is presented, by comparing the oxygen isotopic composition of his tooth enamel bioapatite with isoscape maps of the oxygen isotopic composition of the spring waters. Greece was divided in three zones (North, Central and South Greece) and stable isotope analysis of teeth samples (16) of living donors was conducted. These samples were compared to the isoscape models of δ^18^O of water from the same areas and with the existing equations and data sets of the literature that correlate the oxygen isotope values of the consumed water with the oxygen isotope values of the tooth enamel bioapatite.

The method was implemented in two case studies:

14 human teeth samples were analyzed (that the consumed water during their early years of their life was unknown) and it was possible to confirm or to disproof the locality of the samples.

Furthermore, the methodology was applied to archeological samples of human teeth from Roman Edessa and Medieval Thebes and investigated the effect of the altitude of the water reservoirs and of the consumption of liquids other than water to the isotopic values of ^18^Oen. The equation that relates the ^18^O_enC_ with the ^18^Ow of spring water was used for paleomobility studies of ancient Greek population of Medieval Thebes and Roman Edessa and in the case of Medieval Thebes we detected individuals with oxygen isotopic values very different from the contemporary oxygen isotopic values of spring waters of the areas, which is a strong indication existence of several immigrants. This conclusion is supported by historical sources (Medieval Thebes was ruled by the Franks (1,204–1,311), the Catalans (1,311–1,379), the Navarrese Company (1,379–c. 1,388) and the Florentines (ca.1388–1,460)) while the area was populated by ethnically/culturally diverged people (i.e. Greeks, Jews, Armenians and Albanians).

In my knowledge this methodology is applied to the geographic region of Greece for the first time. Greece is relative very small compared to the regions that this methodology was applied and there are not extreme variations in precipitation and climate that would facilitate the discrimination of the samples versus the geographic regions. Despite the above restrictions, I believe this report adds more insight into the role of stable isotopes in human identification and paleomobility studies.

## Data Availability

All data are available after contacting the author.
